# The Fatality of the Notifiable Diseases

**Published:** 1901-11-16

**Authors:** 


### Hospital Clinics and Medical Progress.

### THE FATALITY OF THE NOTIFIABLE DISEASES.

The experience of medical men, in different places and at different times, must always vary a good deal in regard to the fatality of infectious diseases. Those who practise among the well-to-do are naturally apt to look more lightly upon fevers which, in their fortunate experience, are generally recovered from, than those do who practise in poor neighbourhoods, where often whole families are swept off by them; while, as is well known, the type of disease is subject to considerable modifications from year to year. It is, then, a matter of some interest to extract from the recently issued report of the Local Government Board the fatality rates of the various notifiable diseases during the year 1900, expressing as these do the average case mortality over the whole of the metropolitan area, and in 218 urban districts, a total population of 15,787,963. In this population 540 cases of small-pox were reported during the year, with a fatality of 11·3 per cent. There were 71,283 cases of scarlet fever, with a fatality of 3·4 per cent.; 35,653 cases of diphtheria and membranous croup, with a fatality of 16·9 per cent.; 71 cases of typhus, with a fatality of 25·4 per cent.; 19,947 cases of enteric or typhoid fever, with a fatality of 17·4 per cent.; and a small number of cases entered as continued and relapsing fever, with a fatality of 10 per cent. In regard to all the above diseases the necessity for notification is fairly well recognised and the maladies themselves are sufficiently well defined, so that we may assume that the figures are approximately correct, and may be taken as representing the average fatality of these diseases at the present time among urban populations. Of erysipelas 15,642 cases were notified, with a fatality of 4·5 per cent., a death rate which will seem high or low in proportion as we are accustomed to give the name to all sorts of inflammatory complications of wounds, or to restrict it solely to the more serious forms of apparently "idiopathic" erysipelas. Here we come to the fringe of one great difficulty which complicates the problem of estimating the fatality of diseases, namely, the very different manner in which different medical men use the names by which these maladies are distinguished. When we come to puerperal fever this difficulty is more accentuated. The cases notified numbered 1,266, and of these no less than 704 ended fatally, giving a fatality of 55·6 per cent. In view of these figures there can hardly be any doubt that many cases escaped notification, and that in many instances notification was delayed until a fatal issue, to say the least of it, appeared not unlikely. The fact is that no satisfactory definition of puerperal fever has yet been devised, and we find ourselves faced with the absurdity that while the Act demands notification of "puerperal fever," the Nomenclature of Diseases issued from His Majesty's Stationery Office sixteen years ago, expressly stated that the term "puerperal fever" had been expunged from the Nomenclature, as it still remains.

In addition to this difficulty of nomenclature, the early and complete notification of puerperal fever is probably greatly hampered by the tradition, which still lingers in the teaching, although long since abolished from the practice of certain obstetrical specialists, to the effect that any practitioner who has had to do with puerperal fever should for a somewhat prolonged period hold aloof from midwifery practice. So long as this doctrine is taught, in defiance of the daily example of consulting obstetricians and gynæcologists, who fulfil their appointments, we venture to say, with but small regard to the nature of the preceding case, so long will medical men hesitate to cry "puerperal fever," and to defame their practices by the admission that they are attending such a disease—especially while there is no generally accepted definition of the malady which would not at the same time include cases in which no one would dream of enforcing such a state of quarantine. The acme of oddity, in regard to the lack of relation between notification and death-rate, occurs however under the head of cholera. Here we find that whereas only five cases were notified the deaths amounted to 76! Evidently the medical men who have attended these cases consider that for purposes of registration and for those of notification the word "cholera" has quite different meanings. This anomaly is only a part of the general muddle which has so long obscured the nomenclature of diarrhœal diseases. The College of Physicians have lately endeavoured to clarify this complicated question by the issue of certain recommendations on the subject, but it is to be feared that the proposed reform has not yet made any great advance. When a child has a little diarrhœa its mother is probably told that its stomach is out of order. If baby becomes worse it may be said to have diarrhœa or gastric catarrh, or gastro-enteritis, or muco-enteritis, whatever that is. If it has much pain it has enteritis, and if there is blood in the evacuations it has dysentery, or dysenteric diarrhœa, or colitis; while if there are two or three other children in the same street suffering in the same way its complaint becomes epidemic diarrhœa, or inflammatory diarrhœa, or choleraic diarrhœa, or zymotic enteritis. Again, if it is hot weather, the same complaint becomes summer diarrhœa or cholera nostras, while if the patient dies it becomes cholera. According to the public view a doctor is a fool who lets a child die of diarrhœa, a malady which any old woman thinks herself competent to treat, whereas it is quite excusable for anyone to lose a case of cholera. Thus diarrhœa, choleraic diarrhœa and cholera become mere synonyms for slight, and bad, and fatal cases of the same complaint. All of which has to be remembered when we discuss the case-mortality of diseases.

